# Thyroid cancer surgical indication during pregnancy: Systematic literature review and series of illustrative cases^☆^^☆☆^^☆☆☆^

**DOI:** 10.1016/j.bjorl.2025.101643

**Published:** 2025-05-28

**Authors:** Lucas Albuquerque Chinelatto, Flávio Carneiro Hojaij, Dorival de Carlucci, Claudio Roberto Cernea

**Affiliations:** Universidade de São Paulo (USP), Faculdade de Medicina (FM), São Paulo, SP, Brazil

**Keywords:** Thyroid cancer, Papillary, Thyroid nodule, Pregnancy, Thyroidectomy

## Abstract

**Objective:**

To evaluate and discuss timing possibilities for surgical treatment in thyroid cancer in pregnant women.

**Methods:**

Systematic literature review based on online search at the Literatura Latino-Americana e do Caribe em Ciências da Saúde (LILACS) and National Center for Biotechnology Information (NCBI) databases. Retrospective analysis of thyroidectomies performed in the second trimester of pregnancy by the authors between 1999 and 2019.

**Results:**

The systematic review included nine articles. Their conclusions diverge with respect to the optimal timing of thyroid surgery. The medical literature considers thyroidectomy after safe delivery. The most recent studies are more flexible regarding carrying out this surgery during the second trimester of pregnancy. In the authors’ experience (n = 5), surgical treatment during the second trimester of pregnancy is a good option for more aggressive tumors.

**Conclusion:**

More aggressive cases of thyroid papillary carcinoma can be treated with surgery during the second trimester of pregnancy. Performing the surgery after delivery is safer in the case of less aggressive cancer cases. The decision should consider hospital costs, surgery risks, and patient anxiety in relation to cancer.

**Level of evidence:**

Level II.

## Highlights

• There is no literature consensus about the better time to perform thyroidectomy in pregnant patients with thyroid cancer.
• Surgery in the second trimester should be considered for the most severe cases, such as those that present lymph node metastasis or effective nodule growth within the first weeks of pregnancy.
• In less severe cases, it is safer to perform thyroidectomy after delivery, decreasing the risks related to surgery to the patient and the fetus.

## Introduction

Thyroid cancer is the second most frequently found type of cancer during pregnancy.^1^, ^2^ Its estimated incidence is 14 cases per 100,000 deliveries, with peak incidence in the 25‒30-year age group.^3^, ^4^ Papillary carcinoma is the main histology in this type of cancer.^3^, ^4^ Diagnosis is performed by Fine Needle Aspiration Biopsy (FNAB), which is indicated after finding altered values in routine prenatal examinations, such as those of TSH and free T4, and the manifestation of symptoms is uncommon.^5^

Diagnosis of thyroid cancer during pregnancy brings insecurity and fear to patients,^4^, ^6^, ^7^ which makes updated medical knowledge on the subject fundamental for a correct surgical clinical approach.

The present study reports five cases of surgical approach to thyroid cancer in the second trimester of pregnancy and their respective outcomes. In order to better support the discussion, a systematic literary review was carried out on a chronological perspective. The objective of this study is to evaluate whether there is consensus on the correct time to approach papillary thyroid carcinoma in pregnant patients and discuss the accepted approaches according to the literature.

## Methods

**Fig. 1:**
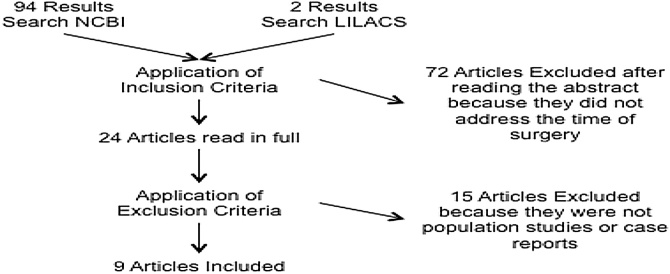
Flowchart of the inclusion and exclusion processes of the studies. Ninety-eight abstracts were analyzed, with final inclusion of nine articles.

The systematic review used the following descriptors in the title or abstract of the publications as a search criterion: “Thyroid” “Surgery” and “Pregnant” [((Thyroid[Title/Abstract]) AND Surgery[Title/Abstract]) AND Pregnant [Title/Abstract]]. An online search was conducted at the Literatura Latino-Americana e do Caribe em Ciências da Saúde (LILACS) and the National Center for Biotechnology Information (NCBI) databases because they are the main databases used in the medical literature. The search was carried out on March 22, 2019 and the abstracts were analyzed for the selection of articles. Inclusion criteria comprised optimal timing to perform thyroidectomy in pregnant patients and outcome evaluation. The following exclusion criteria were adopted: publications other than in English, Portuguese or Spanish; articles not available online; articles other than population studies or case reports. A total of nine articles were analyzed, according to the flowchart in Fig. 1.

A retrospective analysis of thyroidectomies was performed in patients in the second trimester of pregnancy between 1999 and 2019. The series includes cases selected by surgical indication due to the presence of a growing nodule or lymph node metastasis. Intravenous anesthesia with propofol and remifentanil was used in all cases. The option for neck dissection was guided by lymph node involvement. All cases are from the authors’ private practice. Data were processed using Microsoft® Excel® for Mac 2011 14.7.7.

## Results

**Fig. 2:**
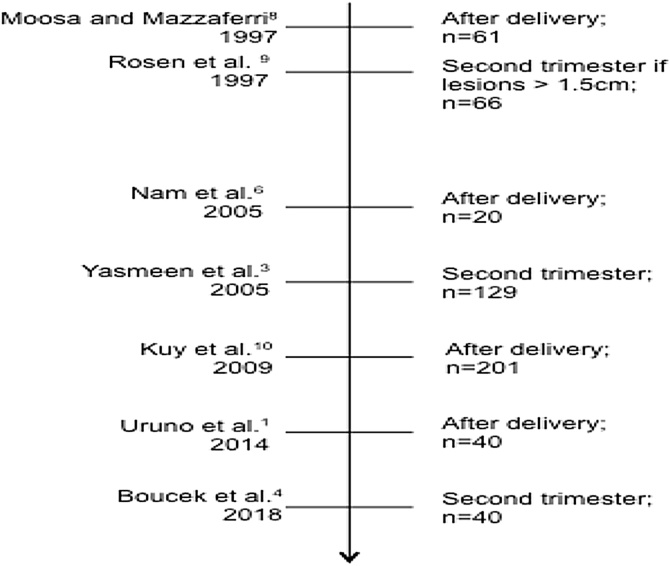
Flowchart with chronological order of publication of the population studies on thyroid cancer in pregnancy. On the right, study conclusion about the time to perform surgery and the study sample (n).

Of the 25 articles read, nine were case reports or population studies. Table 1 shows these articles and their main outcomes. Fig. 2 presents a timeline of the population studies analyzed based on the publication date. It shows that the possibility of indicating surgery in the second trimester of pregnancy, regardless of case severity, is more recent. Even so, there has been no complete change in the indication pattern, as observed in the studies by Kuy et al.^10^ and Uruno et al.^1^ Table 2 presents the studies separated according to their conclusions on the time to indicate surgery, evidencing that constant divergence still exists regarding this topic.

**Table 1:** Systematic review results. Characteristics of the selected studies in chronological order of publication.

Authors	Methodology	Study sample	Time of thyroidectomy: number of patients	Morbidities and deaths	Conclusion
Moosa and Mazzaferri^8^	Cohort of patients treated at the United States Air Force or the Ohio State University Hospitals	61 patients with thyroid cancer during pregnancy	1^st^ trimester: 1	There was no statistical difference between groups	Surgery can be performed after delivery, not delaying it for more than 1-year after delivery
			2^nd^ trimester: 12		
			3^rd^ trimester: 1		
			After delivery: 47		
Rosen et al^9^	Retrospective study: 1982‒1997	66 patients with thyroid cancer during pregnancy	20^nd^ trimester: 14	There was no statistical difference between groups	Perform total thyroidectomy in the 22-weeks when nodule > 1.5 cm.
			After delivery: 37		
Nam et al^6^	Retrospective case study: 1991‒2004	20 patients with thyroid cancer during pregnancy	2^nd^ trimester: 6	There was no statistical difference between groups	Surgery after delivery can generate anxiety, affecting breastfeeding and quality of care
			After delivery: 9		
			Abortions: 5 (non-pathology-related)		
Yasmeen et al^3^	Retrospective cohort study: 1991 and 1999	595, cases of thyroid cancer. 129 cases during pregnancy	2^nd^ trimester: 96	There was no statistical difference between groups	Perform surgery after delivery, not delaying it for more than 1-year as it may increase mortality
			During delivery: 1		
			After delivery: 26		
Kux et al^10^	Retrospective cross-sectional study: 1999‒2005	201 pregnant women compared to 31,155 non-pregnant women	Not specified	There was no statistical difference between groups	Operate cases in the 2^nd^ trimester
Gietka- Czernel et al^11^	Case report ‒ Department of Endocrinology of a hospital in Warsaw, Poland	1 patient at the 21 week of gestation	Emergency thyroidectomy performed in the 2^nd^ trimester	There was no statistical difference between groups	Due to higher costs and longer hospital stay, it is better to operate after delivery
Uruno et al^1^	Retrospective review 1989‒2011	45 patients with thyroid cancer during pregnancy	20^nd^ trimester: 24	There was no statistical difference between groups	Surgery after delivery
			After delivery: 21		
Ismi et al^12^	Case report	1 patient at 23-week of gestation with a neck mass of 3 cm, growing	Total thyroidectomy in the 2^nd^ trimester + Total lymphadenectomy	There was no statistical difference between groups	Operate after delivery. In cases of rapid nodule growth, compressive symptoms, spinal cord or affected lymph node metastases, thyroidectomy should be performed in the 2^nd^ trimester
Boucek et al^4^	International observational cohort. Patients registered between June 2004 and October 2016	40 patients with thyroid cancer during pregnancy 5 excluded for cancer not being well-differentiated	20^nd^ trimester: 29	3 abortions: 1 due to fetal malformation and 2 by maternal desire	Operate in the 2^nd^ trimester. Waiting tox childbirth can increase maternal psychological stress during pregnancy
			After delivery: 6

**Table 2:** Classification of the articles analyzed in the systematic review according to their conclusions regarding best time for surgery.

Second Trimester		After delivery
Any case	Only cases considered serious according to author discretion	
Yasmeen et al^3^	Rosen et al^9^	Moosa and Mazzaferri^8^
Boucek et al^4^	Gietka-Czernel et al^11^	Nam et al^6^
	Ismi et al^12^	Kuy et al^10^
		Uruno et al ^1^

**Fig. 3:**
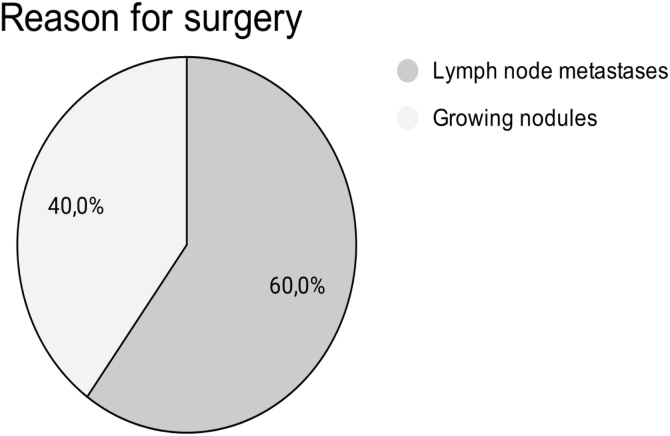
Surgical indication of the series of cases in which the decision was, together with the clinical team, to address cancer during the second semester of pregnancy.

**Fig. 4:**
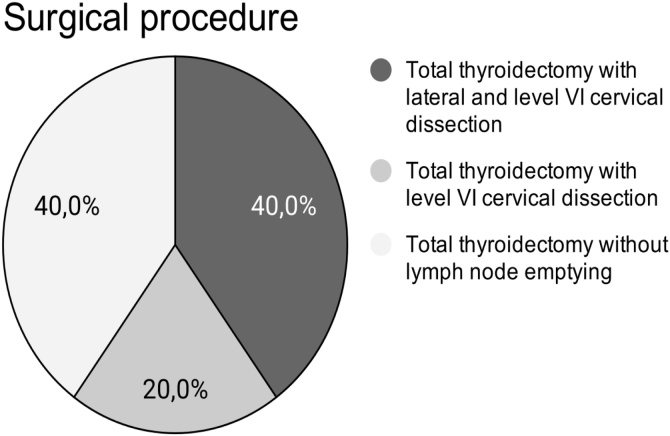
Surgical procedures performed by the authors in their series of cases divided according to the type of cervical dissection performed.

In our experience, we present five case reports surgically addressed during the second trimester of pregnancy, referring to all cases performed by our team at this time of pregnancy (Table 3). As it can be observed in Fig. 3, Fig. 4, surgeries were indicated because of lymph node metastasis or growing nodule. In one of the five cases, the recorded growth was from 1 to 2.5 cm in two months. The decision for surgery in the second trimester was agreed between patient, obstetrician, endocrinologist, and surgeon. No cases presented fetal, anesthetic, or surgical complications. Hypoparathyroidism was observed in all of the cases, being transient in three of them. In order to ensure fetal safety, the fetus was monitored preoperatively, in the immediate postoperative period, in an anesthetic recovery room, as well as twice a day until hospital discharge.

**Table 3:** Case series. Description of surgical cases performed by the authors.

Number of patients		5
Age (years)		25‒38
Follow-up		Minimum of 3 years
Surgery		15‒25 weeks (2^nd^ trimester)
Reason	Growing nodules	2
	Lymph node metastasis	3
Type of Surgery	Total thyroidectomy with lateral level VI cervical dissection	2
	Total thyroidectomy with level VI cervical dissection	1
	Total thyroidectomy without lymph node dissection	2
Delivery		Cesarean section without intercurrences
Obstetric examinations		Normal, pre- and post-surgical intervention
Anesthesia		Intravenous (propofol + remifentanil)
lodine therapy		Starting 3-months after delivery
Evolution		Without intercurrences

The length of hospital staying of the patients ranged from 2 to 4 days, and there were no postoperative complications.

## Discussion

During prenatal care, it is customary to perform thyroid investigation by dosing TSH and free T4 levels, and it is important to analyze them carefully, since normal values are different in pregnancy.^13^, ^14^ If there are changes in these tests or a nodule is found on physical examination, further investigation by Ultrasound (US) and Fine Needle Aspiration Puncture (FNAP) should be conducted.^13^ However, we believe that thyroid nodule investigation should be performed in the pre-gestational period when possible. During the gestational period, the use of scintigraphy with radioactive iodine should be excluded, given the risks for fetal development.^11^, ^13^ Surgical resection is the usual conduct in confirmed cases of papillary thyroid carcinoma; however, the most appropriate time to perform surgery is controversial.^1^, ^6^

Surgery timing can be divided into pregnancy and postpartum. When approached during pregnancy, the optimal indication for surgery is the second trimester, since there is a teratogenic risk in the first trimester and a risk of early delivery in the third trimester.^6^, ^15^ The decision to operate during pregnancy is associated with the effects of human Chorionic Gonadotropin (hCG) on nodule growth due to reactivity of hCG with the TSH receptor.^14^, ^16^ More recent studies have also indicated that most of thyroid cancers in pregnancy present Estrogen Receptors alpha (ER-α), and their growth is therefore stimulated by high estrogen levels.^17^

More recent studies and reviews have shown that there is no difference in mortality and complications, whether maternal or fetal, between operating in the second trimester or after delivery.^18^ Nevertheless, some studies insist on the need to always perform surgery after delivery.^1^, ^6^, ^8^, ^10^

Many studies have reported the option to operate during pregnancy due to the anxiety caused by cases of more aggressive tumors,^2^, ^8^, ^12^, ^13^^,^^19^ as it occurred in the cases herein reported. Aggressive, or locally advanced histology, cervical metastases, compressive symptoms, and rapid nodule growth^13^, ^14^, ^15^, ^18^, ^20^ are the main aggressiveness criteria adopted to indicate surgery in the second trimester, as it occurred in the case of the present study, showing excellent results. However, other authors have used tumor size criteria, ranging from 1.0 to 1.5 cm, as suggestive of greater aggressiveness and surgical indication of patients in the second trimester of pregnancy.^9^, ^21^, ^22^

Another group of authors has always choosen to indicate surgery in the second trimester.^3^, ^4^, ^9^, ^11^ Such decision aims to reduce maternal anxiety^23^ and promote better maternal care and breastfeeding after delivery.^9^, ^20^ In any case, it should be considered that surgical intervention in the second trimester of pregnancy has higher costs (approximately USD 300 extra) in addition to a one-day increase in length of hospital stay.^10^

When a choice is made for surgical intervention in the second trimester of pregnancy, there is consensus that some extra care should be taken. Choosing a high-volume surgeon, for example, means a better prognosis.^3^, ^4^, ^9^, ^11^ Anesthesia should also be considered, and some groups have suggested the use of local anesthesia with cervical plexus block associated with the use of benzodiazepines and short-term opiates.^7^, ^19^ In our experience, the use of intravenous anesthesia with propofol and remifentanil did not present maternal or fetal complications in any of the cases.

## Conclusion

There is no consensus on the correct time to perform thyroidectomy in pregnant patients with thyroid cancer. Although the review does not bring innovations, it provided collection of clearer data for the definition of conducts.

Operating the most severe cases in the second trimester is an option, as shown in our case series and described in the literature. We understand that more severe cases are those that present lymph node metastasis or effective nodule growth in the first weeks of pregnancy. In less severe cases, it is safer to perform thyroidectomy after delivery, and the risks to the patient and the fetus, patient anxiety, and costs of hospital stay should also be considered.

## Financial support

None.

## Declaration of competing interest

The authors declare no conflicts of interest.
